# Expression of microRNA‐like RNA‐2 (*Fgmil‐2*) and *bioH1* from a single transcript in *Fusarium graminearum* are inversely correlated to regulate biotin synthesis during vegetative growth and host infection

**DOI:** 10.1111/mpp.12859

**Published:** 2019-08-06

**Authors:** Mao‐Wei Guo, Peng Yang, Jing‐Bo Zhang, Gang Liu, Qing‐Song Yuan, Wei‐Jie He, Jun‐Na Nian, Shu‐Yuan Yi, Tao Huang, Yu‐Cai Liao

**Affiliations:** ^1^ Molecular Biotechnology Laboratory of Triticeae Crops Huazhong Agricultural University Wuhan 430070 People's Republic of China; ^2^ College of Plant Science and Technology Huazhong Agricultural University Wuhan 430070 People's Republic of China; ^3^ College of Life Science and Technology Huazhong Agricultural University Wuhan 430070 People's Republic of China

**Keywords:** biotin biosynthesis, *Fusarium graminearum*, micro‐like RNAs, mycotoxin, post‐transcriptional regulation, small RNA sequencing

## Abstract

MicroRNA‐like RNAs (milRNAs) post‐transcriptionally down‐regulate target genes. We investigated *Fusarium graminearum* (*Fg*) milRNA expression during fungal vegetative growth and infection of wheat. Small RNA sequencing identified 36 milRNAs from *Fg*, one of which, *Fgmil‐2*, had >100 transcripts per million in conidia, mycelia and infected wheat, with the highest expression in conidia and the lowest expression in colonized wheat tissue. *Fgmil‐2* displays perfect homology to the 3ʹ‐untranslated region (3ʹ‐UTR) of an *FgbioH1* messenger RNA that is involved in biotin biosynthesis. Poly(A) polymerase‐mediated rapid amplification of cDNA ends combined with sequencing analysis demonstrated that cleavage at a specific site by FgDicer2 in the 3ʹ‐UTR of *FgbioH1* transcripts generated the *Fgmil‐2* precursor with a typical hairpin structure. Deletion of *FgbioH1* or *FgDicer2* genes abolished *Fgmil‐2* biogenesis. *FgbioH1* had an inversely correlated pattern of expression to that of *Fgmil‐2* and *FgDicer2*. Deletion of *FgbioH1* also showed that it is required for mycelial growth, virulence, mycotoxin biosynthesis and expression of biotin‐dependent carboxylase genes. This study reveals in *Fg* a novel mode of inversely correlated post‐transcriptional regulation in which *Fgmil‐2* originates from its own target transcript, *FgbioH*, to govern biotin biosynthesis.

The ascomycete fungal phytopathogen *Fusarium graminearum* (*Fg*) is the globally distributed causal agent of fusarium head blight (FHB) of wheat, costing billions of dollars in lost agricultural productivity (Goswami and Kistler, 2004; Qu *et al*., 2008). *Fg* produces trichothecene mycotoxins in infected grains, which are highly toxic to humans and domestic animals (Pestka and Smolinski, 2005). Elucidation of the regulatory mechanisms of *Fg* pathogenesis is thus essential for FHB control strategies. To this end, we sought to identify the potential roles of microRNAs (miRNAs) in fungal pathogenesis. miRNAs or miRNA‐like RNAs (milRNAs) post‐transcriptionally down‐regulate genes in eukaryotes (Bartel, 2004; Torresmartínez and Ruizvázquez, 2017). In animals, primary transcripts (pri‐miRNAs) are initially cleaved by the ribonuclease (RNase) III Drosha, then transported to the cytoplasm and cleaved into *c*.22 nt (nucleotide) miRNA duplexes by Dicer, a cytoplasmic RNase III (Court *et al.*, 2013; Havens *et al.*, 2014; Kurzynska‐Kokorniak *et al.*, 2015).

In contrast, fungal miRNAs are produced by at least four different pathways, but lack Drosha (Lee *et al.*, 2010). In *Fg*, *FgDicer2* was shown to be involved in hairpin‐induced gene silencing during vegetative growth (Chen *et al.*, 2015). Identification of the targets of miRNA regulation is also important for understanding the mechanisms of *Fg* development and pathogenesis. In particular, biotin, a highly conserved vitamin cofactor for biotin‐dependent carboxylases (Pirner and Stolz, 2006), has been shown to be essential for development in yeast and other eukaryotes. Furthermore, biotin plays a role in the epigenetic regulation of gene expression and cell signalling in mammals (Zempleni *et al.*, 2009).

In this study, RNA deep sequencing identified 36 milRNAs in *Fg*, one of which, *Fgmil‐2*, displayed a differential pattern of high expression in conidia and mycelia but substantially lower expression during infection of wheat. We found that this pattern of expression was inversely correlated with transcription of *FgbioH1* mRNA, the translated product of which catalyses the hydrolysis of pimeloyl‐[ACP] methyl ester to pimeloyl‐[ACP], an essential step in the biotin biosynthesis pathway. To explore the roles of *FgbioH1* and *Fgmil‐2* in the infection process, we generated deletion mutants of *FgbioH1* and *FgDicer2*. In the absence of *Dicer2*, only *FgbioH1* mRNAs were produced. In the absence of *FgbioH1*, fungal growth, successful infection of spikelets and mycotoxin synthesis all decreased. We thus propose that a *Dicer‐2*‐mediated post‐transcriptional regulatory switch determines the spatiotemporal and inversely correlated expression of *Fgmil‐2* and *bioH1* from a single transcript during the transition from vegetative growth to host colonization by *Fg*. These results provide foundational insights into the mechanisms and regulation of milRNA biogenesis as well as a putative role for biotin in fungal vegetative growth and infection of plants.

To systematically identify *Fg* milRNAs transcribed during vegetative growth and infection of wheat, two small RNA (sRNA) libraries were constructed from conidia and from mycelia of *Fg* strain 5035, a deoxynivalenol (DON)‐producing strain isolated from a spike of scabby wheat in China (Method S1, see Supporting Information), and four sRNA libraries were constructed from wheat spikes at 0, 48, 72 and 96 h after inoculation (hai) with *Fg*. The total number of clean reads from each sample that matched the *Fg* genome are listed in Table S1 (see Supporting Information). sRNA length distributions from each sample and 5' terminal base preference are shown in Figs S1 and S2 (see Supporting Information). The as‐of‐yet unexplained variation in sRNA length distribution and 5' terminal base preference among the six libraries may be common among fungi, as similar phenomena have been reported in other fungal species (Lee *et al.*, 2010).

Prediction of hairpin structures and free energy of folding by MIREAP software identified 36 candidate milRNAs among the *Fg* sRNA reads with a free energy of folding cut‐off value of −58.83 kcal/mol (Weiberg *et al.*, 2013; Table S2, see Supporting Information). Among these milRNAs, 31 were expressed in conidia, and 23 of these were exclusively expressed in this developmental stage (Fig. 1A). In contrast, five of the ten milRNAs detected in mycelia were specific to that stage. In wheat spikes, six milRNAs were detected, all of which were also found in conidia. Expression levels of these milRNAs varied widely, with most of them having low or no expression in conidia, mycelia or infected wheat (Fig. S3, see Supporting Information). Three milRNAs, *Fgmil‐1*, *‐2* and *‐3*, had relatively high expression levels in at least one of the tissues sampled (Figs 1A and S3) at a cut‐off value of >100 transcripts per million (TPM) (Weiberg *et al.*, 2013).

**Figure 1:**
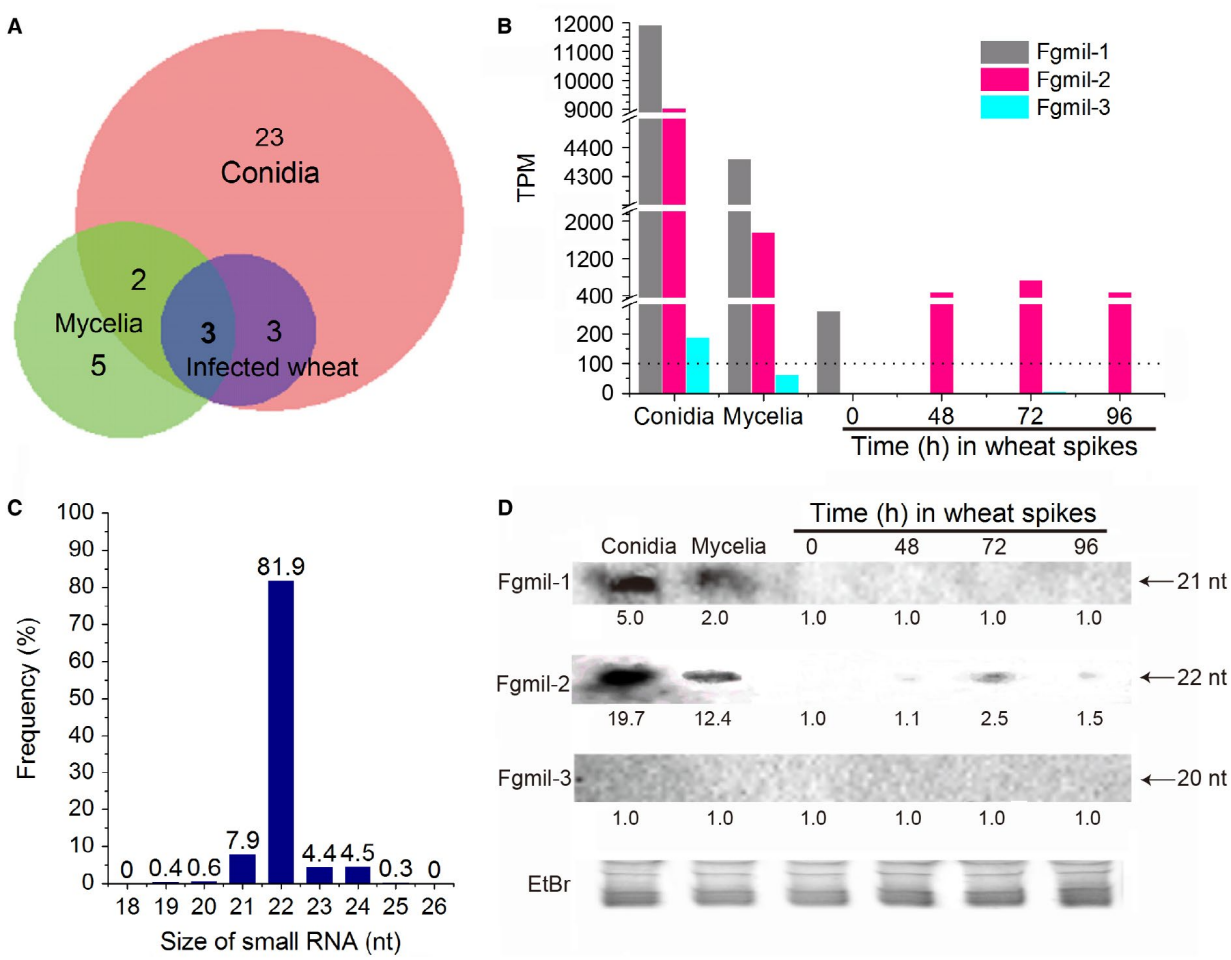
Expression patterns of milRNAs in *Fusarium graminearum*. (A) The Venn diagram illustrates the overlap of milRNA expression in conidia, mycelia and infected wheat. (B) Expression levels of three milRNAs selected based on 100 transcripts per million (TPM) as a cut‐off value. TPMs from fungal conidia and mycelia and wheat spikes at 0, 48, 72 and 96 h after inoculation. (C) Distribution frequency of different nucleotide sizes ranging from 18 to 26 nt in *Fgmil‐2*. (D) Northern blot detection of mature milRNA levels. Thirty micrograms of total RNAs were separated on a 15% polyacrylamide gel, then transferred to nylon membranes, and digoxigenin (DIG)‐labelled oligonucleotides antisense to the respective milRNAs were used as probes. The ethidium bromide (EtBr)‐stained denaturing gel in the bottom panel shows equal loading of RNA. The number below each panel indicates the intensity of the blot band, in which the ‘time 0’ sample was arbitrarily designated as the baseline of expression for comparisons of changes in expression over time. nt, nucleotide.

Only *Fgmil‐2* was found at >100 TPM in conidia, mycelia and wheat at 48, 72 and 96 hai (no signal detected at 0 hai) (Fig. 1B), whereas the other two milRNAs were not detectable at all during wheat colonization (at 48, 72 and 96 hai). *Fgmil‐2*, having the characteristic milRNA features of 22 nt length (Fig. 1C) and a 99.8% preference for a U at the 5' terminus, displayed a distinct pattern of differential expression between fungal vegetative growth and host colonization, i.e. the highest expression (9033 TPM) was observed in conidia, with a 5.2‐fold decline in mycelia (1753 TPM), and 12.3‐ to 18.8‐fold reduction in wheat at 48, 72 and 96 hai (479–735 TPM). Further northern blot experiments confirmed that *Fgmil‐1* and *Fgmil‐2* were both expressed at high levels in conidia and mycelia while *Fgmil‐3* was undetectable in these samples. In contrast, only *Fgmil‐2* was detected in infected wheat spikes, with the highest expression at 48 hai (Fig. 1D and Table S3, see Supporting Information), thus confirming the reliability of the sRNA sequencing results. Interestingly, *Fgmil‐2* was found to have the same sequence as *Fg‐milRNA‐5* identified by Chen *et al.* (2015) in mycelia of *Fg* strain HN9‐1. The transcript of origin and its expression in conidia and infected plants were not examined in that work. These patterns of expression and milRNA characteristics led us to more closely examine the origin and role of *Fgmil‐2* in *Fg* development.

Unexpectedly, no predicted target sequences aligning with *Fgmil‐2* were identified in wheat or other crops. This finding prompted us to search the *Fg* genome for potential precursor mRNAs. We found that *Fgmil‐2* aligned with the 3ʹ‐UTR of FGSG_01659, within which NCBI blastp revealed a pimeloyl‐[ACP] methyl ester carboxylesterase (bioH) domain (Fig. S4, see Supporting Information). This *Fg* gene was thus designated *FgbioH1*. Given that Dicer2 is involved in microRNA biogenesis in *Fg* (Chen *et al.*, 2015), we generated *FgbioH1* and *FgDicer2‐*deletion mutant strains, Δ*FgbioH1* and Δ*FgDicer2* (Table S3, see Supporting Information), to experimentally verify that *Fgmil‐2* is encoded within *FgbioH1*. After confirmation by Southern blot of successful deletion at both loci (Fig. S5, see Supporting Information), Δ*FgDicer2*, Δ*FgbioH1* and wild‐type (WT) *Fg* strain 5035 were subjected to sRNA HiSeq platform sequencing. While WT 5035 was shown to contain 1495 TPM of *Fgmil‐2*, no *Fgmil‐2* transcripts were detected in the Δ*FgDicer2* or Δ*FgbioH1* strains (Fig. 2A). These results indicate that both *FgDicer2* and *FgbioH1* genes are essential for *Fgmil‐2* biogenesis.

**Figure 2:**
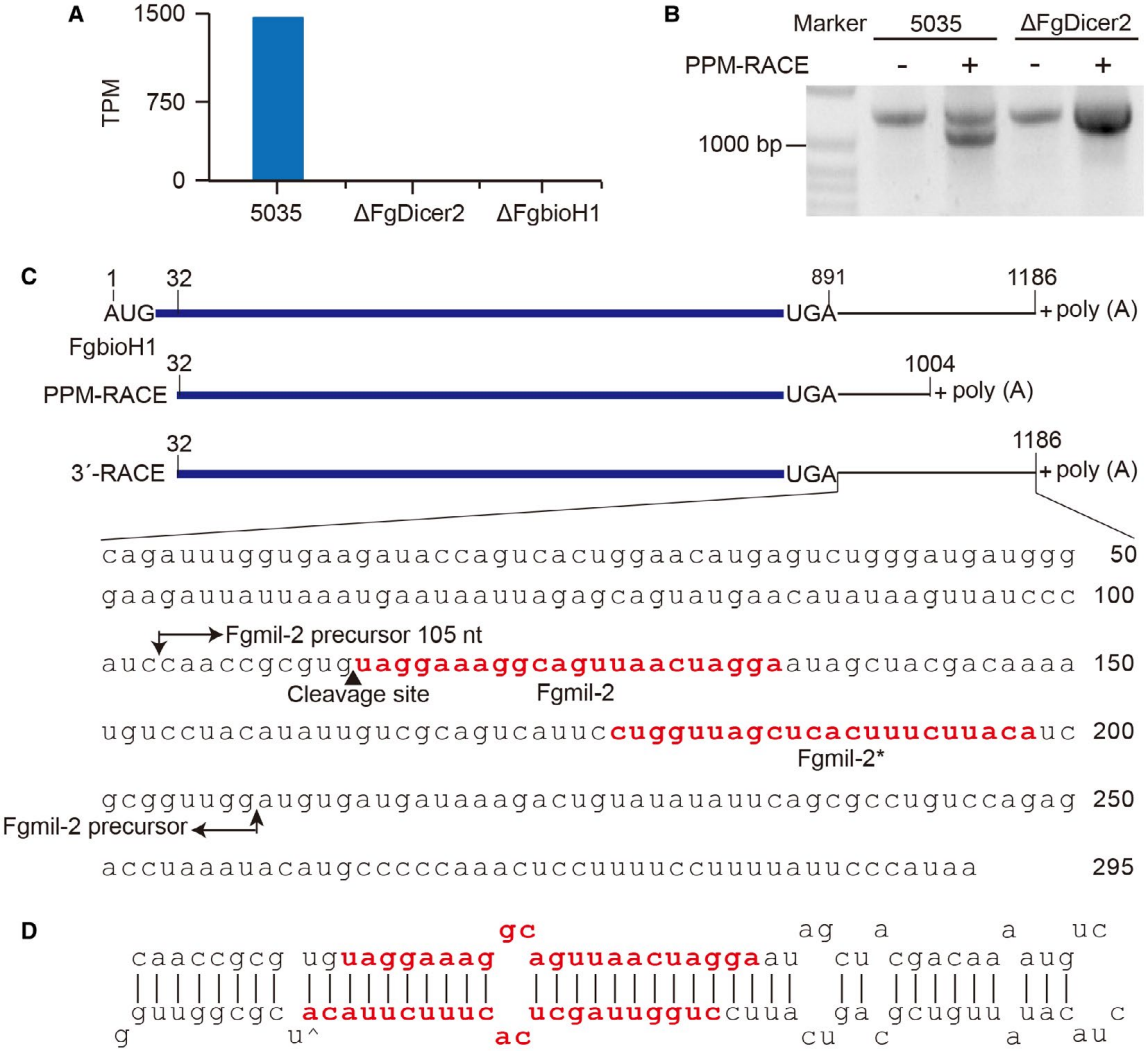
Identification of origin and cleavage site of *Fgmil‐2*. (A) Expression levels of *Fgmil‐2* in wild‐type (WT) strain 5035, *FgDicer2* gene‐deletion mutant strain Δ*FgDicer2* and *FgbioH1* deletion mutant Δ*FgbioH1*. Histograms represent transcripts per million (TPM). (B) Products of *FgbioH1* transcripts amplified by poly(A) polymerase‐mediated rapid amplification of cDNA ends (PPM‐RACE) and 3ʹRACE of WT strain 5035 and mutant strain Δ*FgDicer2*. +, with PPM‐RACE; –, without PPM‐RACE. (C) Schematic diagram of *FgbioH1* mRNA and its 3ʹ‐UTR sequence. Numbers above the panel are nucleotide positions of *FgbioH1* mRNA. Sequence between long arrows represents predicted *Fgmil‐2* precursor (105 nt) within 3ʹ‐UTR sequence. Cleavage site is determined by PPM‐RACE. Red nucleotides indicate *Fgmil‐2* mature sequence and its complementary chain *Fgmil‐2* ^★^. (D) Schematic diagram showing a secondary hairpin structure of *Fgmil‐2* precursor (105 nt), with thermodynamic: Δ*G* = −62.20 kcal/mol.

To obtain the sequence from which *Fgmil‐2* is generated, poly(A) polymerase‐mediated rapid amplification of cDNA ends (PPM‐RACE) was used to amplify transcripts derived from the 3ʹ‐UTR of *FgbioH1* mRNAs lacking poly(A) tails. Total RNAs were isolated from Δ*FgDicer2*, Δ*FgbioH1* and WT strain 5035 and used for PPM‐RACE and 3ʹRACE (Table S3, see Supporting Information). Each RNA sample was divided into two parts: one part was used for PPM‐RACE and 3ʹRACE, and the other was used for 3ʹRACE only.

Simultaneous PPM‐RACE/3ʹRACE of WT 5035 RNA generated two DNA fragments with different sizes, whereas 3ʹRACE of WT 5035 produced only one larger fragment (Fig. 2B), indicating the presence of two species of *FgbioH1* transcripts in the WT strain. In contrast, PPM‐RACE/3ʹRACE and 3ʹRACE alone of Δ*FgDicer2* RNAs produced only one large DNA fragment, indicating the presence of only one type of *FgbioH1* transcript in the Δ*FgDicer2* strain. These results confirm the presence of two types of *FgbioH1* transcripts in WT strain 5035: large integral *FgbioH1* transcripts and small Dicer2‐cleaved *FgbioH1* transcripts. However, only the large uncleaved *FgbioH1* transcripts were observed in Δ*FgDicer2*, indicating that *FgDicer2* participates in generating smaller *FgbioH1‐*derived transcripts.

To determine the precise location of the *Fgmil‐2* sequence within the *FgbioH1* transcript, we examined the PPM‐RACE and 3ʹRACE amplicons. Sequence analysis revealed that the large fragments from strains WT 5035 and Δ*FgDicer2* (Fig. 2B) were all identical 1154 bp *FgbioH1* gene sequences with poly(A) tails, whereas the small fragment was a 972 bp subfragment of *FgbioH1* with a stretch of poly(A), indicating a 182 bp size difference (Fig. 2C). The 3ʹ‐UTR of the *FgbioH1* mRNA was 295 nt and contained the *Fgmil‐2* precursor (105 nt), as predicted by MIREAP software; thus, the *Fgmil‐2* precursor starts 103 nt downstream of the UGA stop codon. Sequence analysis showed a guanine base at the 3ʹ end of the PPM‐RACE‐derived small fragment, indicating that the FgDicer2 cleavage site is located between guanine (113) and uracil (114) to generate the *Fgmil‐2* milRNA (Fig. S6, see Supporting Information). The *Fgmil‐2* precursor has a typical stem‐loop structure and a free energy of folding of −62.20 kcal/mol (Fig. 2D). Taken together, these results demonstrate that the *Fgmil‐2* precursor is located in the 3ʹ‐UTR of *FgbioH1* mRNA, from which *Fgmil‐2* is generated by FgDicer2 cleavage.

Typically, miRNAs or milRNAs are located within protein‐coding regions (intragenic miRNAs) and target other genes for down‐regulation (Baskerville and Bartel, 2005; Rodriguez *et al.*, 2004). Recently, a primate‐specific exonic miR‐198 hairpin was identified in the 3ʹ‐UTR of *FSTL1* (*Follistatin‐like 1*) mRNA, for which Drosha served as a transcriptional switch by cleavage of the miRNA hairpin, ultimately generating two alternative gene products from a single transcript (Ha and Kim, 2014; Sundaram *et al.*, 2013). To our knowledge, no study has reported finding two such transcriptional products generated from a single mRNA in fungi, where Drosha is absent.

To determine the impact of *FgDicer2* on *FgbioH1*, we compared the relative expression levels of these two genes in conidia, mycelia and infected wheat at 24, 48, 72, 96 and 120 hai in both WT 5035 and Δ*FgDicer2* strains. *FgDicer2* was most highly expressed in WT conidia but sharply declined in mycelia, while its lowest expression was during the infection process (Fig. 3A). Conversely, *FgbioH1* expression was lowest in WT conidia and highest during the infection of wheat, with intermediate expression levels in mycelia. Expression of these two genes is thus inversely correlated. Moreover, in the Δ*FgDicer2* strain the *FgbioH1* gene showed high constitutive expression across all the samples and timepoints. These results strongly indicated that FgDicer2 negatively regulated *FgbioH1* expression, and that functional loss of the *FgDicer2* gene resulted in constitutive expression of *FgbioH1*.

**Figure 3:**
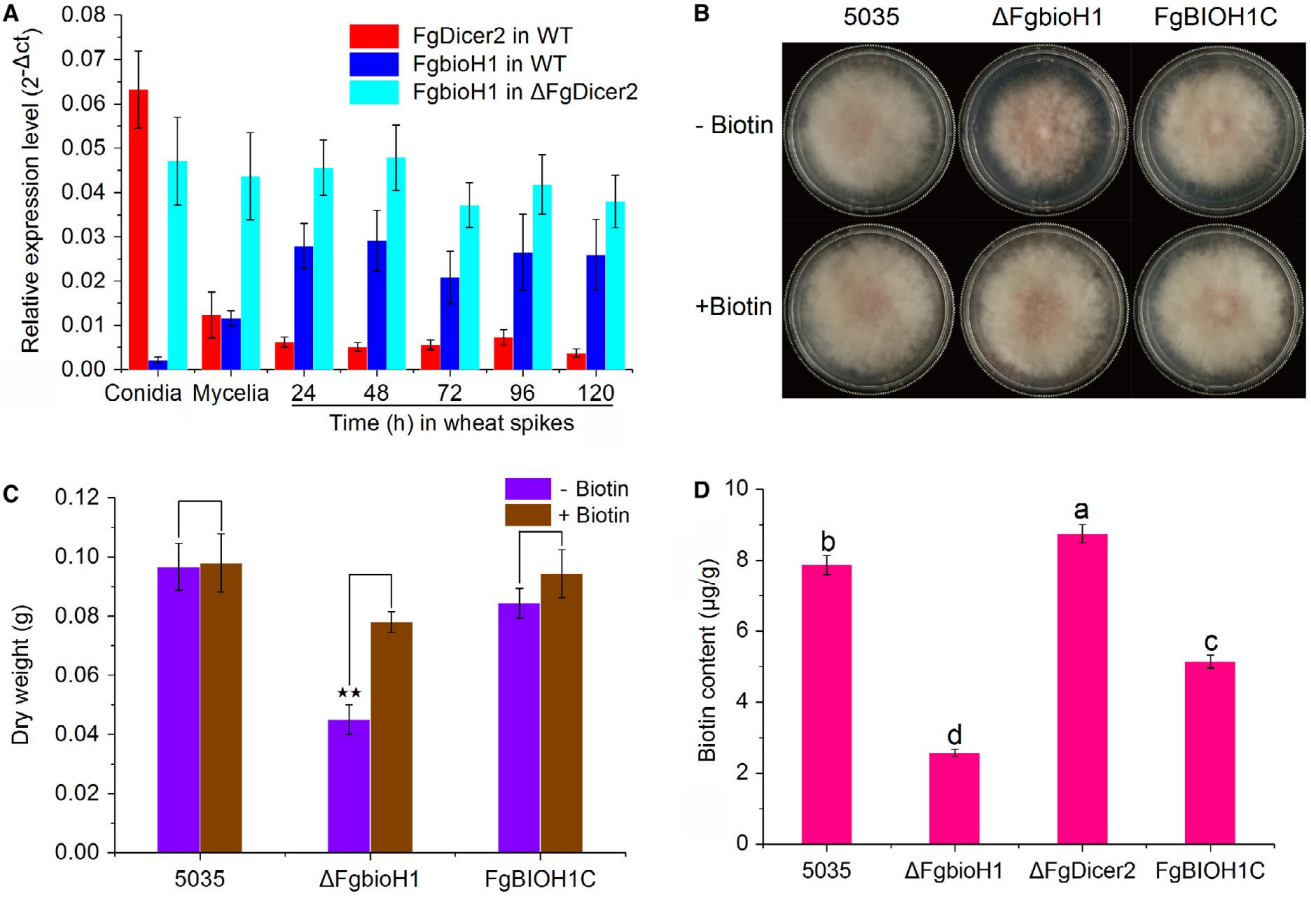
Expression pattern, mycelial growth, biomass and biotin content of different *Fusarium graminearum* strains. (A) Expression levels of *FgDicer2* and *FgbioH1* genes of different *F. graminearum* strains during their vegetative growth and infection of wheat. Red columns and blue columns represent relative mRNA expression levels of *FgDicer2* and *FgbioH1* genes, respectively, in wild‐type (WT) strain 5035, while the cyan columns represent mRNA expression levels of *FgbioH1* genes in Δ*FgDicer2*. Line bars denote standard errors of three biological replicates. (B) Mycelial growth of *F. graminearum* strains WT 5035, Δ*FgbioH1* and complementation strain *FgBIOH1C* on minimal medium (MM) in the absence and presence of biotin (20 ng/mL) for 5 days. (C) Mycelial biomass (dry weight) of three strains described in (B). (D) Biotin content of the three strains in (B) and Δ*FgDicer2*. Experiment was repeated three times. Line bars denote standard errors of three biological replicates (^★★^ *P* < 0.01). Different letters represent a significant difference at *P* < 0.05.

We thus propose that *FgDicer2* plays a decisive role in post‐transcriptional regulation of *FgbioH1* and displays a development‐dependent expression pattern (Fig. 3A) that is perfectly consistent with that of *Fgmil‐2* (Fig. 1). FgDicer2 cleaves the *Fgmil‐2* hairpin structure in the *FgbioH1* 3ʹ‐UTR, causing the mRNA to become vulnerable to degradation by other ribonucleases. Furthermore, the 3ʹ poly(A) tail is an important determinant of translational efficiency. Thus, *FgDicer2*‐deletion clearly correlates with increased *FgbioH1* expression. This activity by FgDicer2 differs from its homologues in that Drosha or Dicer enzymes canonically cleave milRNAs that target other gene sequences for regulation; in those cases Drosha or Dicers indirectly regulate the target genes, whereas FgDicer2 directly effects *bioH1* expression because *Fgmil‐2* targets the same transcripts from which it is generated.

To determine the role of *FgbioH1* in fungal growth and biotin synthesis, we cultured three *Fg* strains, WT 5035, Δ*FgbioH1* and an *FgbioH1*‐complemented mutant *FgBIOH1C* (Fig. S5 and Table S3, see Supporting Information) in the presence and absence of biotin. Deletion of *FgbioH1* significantly reduced mycelial growth and biomass in media (Fig. 3B,C), resulting in a dry weight (DW) of 0.045 g for Δ*FgbioH1*, which was 53% lower (*P* < 0.01) than WT 5035 (DW = 0.097 g); *FgBIOH1C* restored fungal growth and biomass to levels comparable with WT (Fig. 3B,C). To verify that the reduced mycelial growth was due to the lack of biotin, Δ*FgbioH1* was cultured in media supplemented with biotin. In the presence of biotin, Δ*FgbioH1* growth and biomass (DW = 0.078 g) were restored to levels comparable with (i.e. not significantly different from) the WT. Thus, biotin biosynthesis depends on *FgbioH1* and is required for vegetative growth of *Fg*.

In order to compare biotin production among WT 5035, Δ*FgbioH1*, Δ*FgDicer2* and *FgBIOH1C*, HPLC was used to measure biotin in samples of each strain (Fig. 3D). WT strain 5035 produced 7.87 μg/g (dry weight) biotin, whereas Δ*FgbioH1* had 2.58 μg/g, a 67% reduction (*P* < 0.01) relative to WT. The *FgBIOH1C* complementation strain produced 5.15 μg/g biotin, while Δ*FgDicer*2 produced 8.75 μg/g, a significant (*P* < 0.05) 11% increase over production in WT. These results indicate that the *FgbioH1* gene is a predominant contributor to biotin biosynthesis, while *FgDicer*2 down‐regulates biotin biosynthesis, the deletion of which increases biotin content in *Fg*. Notably, biotin synthesis was not completely abolished by *FgbioH1* deletion. This phenomenon is likely attributable to functional redundancy by another *bioH*, FGSG_03039, with 38% amino acid similarity to *FgbioH1*, but lacking homology to the *Fgmil‐2* 3ʹ‐UTR sequence (Fig. S4).

*Fgmil‐2* accumulated to its highest level in resting conidia and so we are inclined to speculate that this relationship may be part of a regulatory state necessary for maintaining low biological activity in conidia. In *Aspergillus niger*, resting conidia were found to exhibit a low level of respiratory metabolism in aqueous suspension (Novodvorska *et al.*, 2016). However, regardless of low metabolic activity that may or may not also occur in *Fg* conidia, *Fgmil‐2* comprises the predominant *FgbioH1* transcript species and biotin does not appear to be required for biomass production and mycelial growth until germination and subsequent host colonization. As conidia germinate to form mycelia, many enzymes are synthesized that require biotin as a cofactor to execute their function; at this point, *FgbioH1* transcripts accumulate to higher levels to ensure adequate biotin synthesis (Fig. 3A). Expression of either form of the original transcript therefore depends on the functional requirements of each *Fg* developmental stage (Figs 1 and 3).

To further characterize the role of the *FgbioH1* gene in host colonization *in planta*, we inoculated wheat with Δ*FgbioH1*, *FgBIOH1C* and WT 5035, and compared the differences in virulence between the strains. We found that WT 5035 infected 29% of the spikelets at 14 dai, similar to that of *FgBIOH1C* (Fig. 4A,B)*.* In contrast, Δ*FgbioH1* showed an 8% spikelet infection rate, a 78% reduction compared to WT. These results demonstrate that loss of *FgbioH1* function results in a substantially decreased ability to colonize plant hosts. As fungal hyphae extend into plant tissues, biotin‐dependent enzymes deplete available biotin, and to subsequently accommodate this need the highest expression of *bioH1* transcripts was then observed.

**Figure 4:**
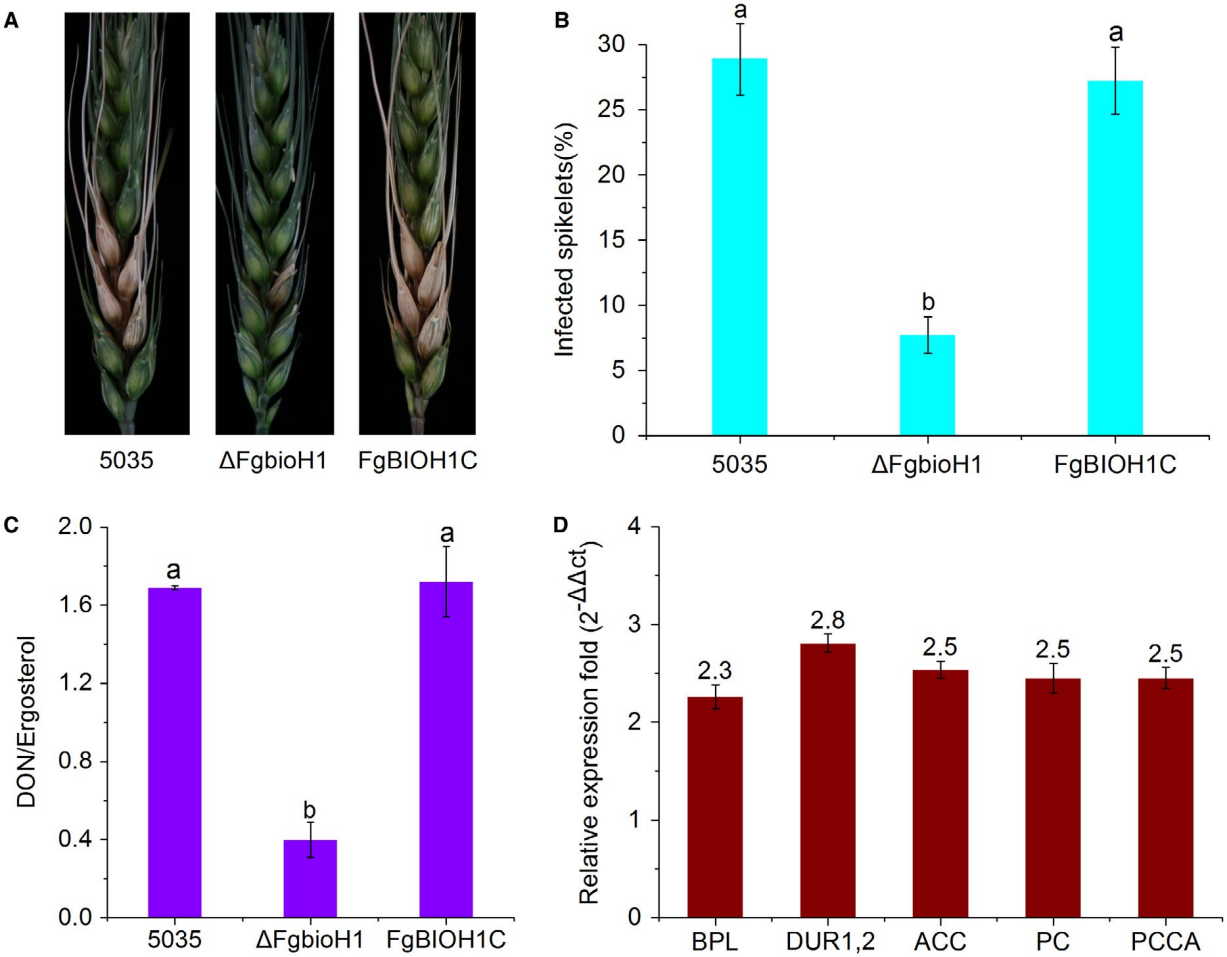
Impacts of *FgbioH1* on virulence, mycotoxin biosynthesis and gene expression. (A) Representative wheat spikes 14 days after inoculation (dai) with *Fusarium graminearum* wild‐type (WT) strain 5035, Δ*FgbioH1* and complementation strain *FgBIOH1C*. (B) Percentage of infected wheat spikelets at 14 dai. Percentages were calculated as means ± SD for each strain. Different letters indicate significant difference at *P* < 0.05. (C) Contents of mycotoxin deoxynivalenol:ergosterol. Data from three biological replicates are averages ± standard errors. Different letters indicate a significant difference at *P* < 0.05. (D) Fold changes in transcript levels from five genes in Δ*FgbioH1* relative to that of WT. Histograms represent fold increase in transcripts from five genes *BPL*, *DUR1,2*, *ACC*, *PC* and *PCCA* that use biotin as a cofactor. Line bars denote standard errors of three biological replicates.

Given the importance of up‐regulating *FgbioH1* expression for successful host colonization, it was also important to test if this gene participated in mycotoxin biosynthesis, an important component of pathogenesis. We evaluated mycotoxin production in the Δ*FgbioH1*, *FgBIOH1C* and WT 5035 strains by calculating their DON:ergosterol ratios. The Δ*FgbioH1* strain showed a significantly lower DON:ergosterol ratio (0.45) than WT 5035 (1.69), a 47% reduction (Fig. 4C). Thus, the *FgbioH1* gene is also required for biosynthesis of DON in *Fg* at levels consistent with successful colonization of wheat.

To probe if *FgbioH1* expression also affects the expression of biotin‐dependent carboxylase‐encoding genes in *Fg*, we used qPCR assays to compare the transcriptional levels of five biotin‐dependent carboxylase genes between the WT and Δ*FgbioH1* strains. From studies in humans and yeast showing regulation by or dependence on biotin as a co‐factor, we selected *BPL* (FGSG_08329), urea amidohydrolase *DUR1,2* (FGSG_10913), an acetyl‐CoA carboxylase (ACC) (FGSG_06580), pyruvate carboxylase (PC) (FGSG_07073) and a propionyl‐CoA carboxylase α subunit (PCCA) (FGSG_08688) (Solórzano‐Vargas *et al.*, 2002) (Table S3, see Supporting Information). Expression levels of all five genes assayed in the Δ*FgbioH1* strain were increased 2.3‐ to 2.8‐fold compared with that of the WT strain 5035 (Fig. 4D). Thus, *FgbioH1* expression decreases expression of other biotin‐dependent carboxylase‐type genes in *Fg*, similar to what has been reported in yeast (Pirner and Stolz, 2006). Down‐regulation of carboxylase genes may directly impact fatty acid biosynthesis and gluconeogenesis, which are essential for primary metabolism (Magliano *et al.*, 2011).

Recently Zhang *et al. *(2016) showed that during *Fg* infection the *bioH1* gene displayed enhanced expression whereas, in contrast, biotin‐dependent enzymes such as DUR1,2, PC and PCCA all had reduced expression compared to their expression in mycelia. This study is in agreement with our results in that both demonstrate an important function for the *bioH1* gene during plant pathogenesis. Importantly, the inversely correlated expression pattern of *Fgmil‐2* and *FgbioH1* transcripts revealed by this study provides insight into the post‐transcriptional regulation governing biotin biosynthesis during fungal colonization of plants.

In addition, this study reveals the sub‐pathway branch for production of the intermediate compound pimeloyl‐[ACP] in *Fg*. The biotin biosynthesis pathway was originally proposed based on studies in *Escherichia coli* because of a dearth of studies regarding biotin biosynthesis in fungi (Fig. S7). In *E. coli*, three sub‐pathways were found for the synthesis of the two intermediate compounds, pimeloyl‐[ACP] and pimeloyl‐CoA, which are substrates for subsequent reactions. Two *bioH* genes in *Fg*, *FgbioH1* and *FgbioH2* are both likely involved in the same sub‐pathway: both genes encode proteins predicted to catabolize the hydrolysis of pimeloyl‐[ACP] methyl ester to form pimeloyl‐[ACP]. Analysis of the *Fg* genome sequence indicated that *Fg* does not carry the genes involved in the other two sub‐pathways (*bioI* and *bioW*). Thus, the biosynthesis of pimeloyl‐[ACP] is the only sub‐pathway in *Fg* to synthesize this intermediate compound for subsequent biotin production. This study can serve as the foundation for further characterization of the regulatory mechanisms for genes in the biotin biosynthesis pathway in fungi.

In conclusion, we show that a microRNA‐like RNA in *Fg*, *Fgmil‐2*, was derived from the 3ʹ‐UTR of an *FgbioH1* messenger RNA and that the Dicer2‐dependent biogenesis of *Fgmil‐2*, through truncation of *FgbioH1* transcripts, led to *FgbioH1* degradation and subsequent down‐regulation. Inversely correlated expression of *Fgmil‐2* and *FgbioH1* from the single *bioH1* transcript was dependent on developmental stage, while *Fgmil‐2* had the highest expression in resting conidia and the lowest expression during infection of wheat, in contrast to *FgbioH1* expression, which was lowest in conidia and highest during infection. *FgbioH1* is involved in the biosynthesis of biotin, which is required for vegetative growth, virulence, mycotoxin biosynthesis and expression of various genes in *Fg*. This study provides insight into the mechanisms of milRNA biogenesis and regulation in pathogenic fungi during development and interaction with host plants. Given the essential nature of biotin for *Fg* development, *FgbioH1* may serve as a target for RNA interference to control FHB and mycotoxins in agricultural production.

## Supporting information


**Fig. S1** Relative proportions of total sRNA sequences in six samples of *Fusarium graminearum* (*Fg*) and wheat. (A) Conidia. (B) Mycelia. (C) Wheat spikes at 0 h after inoculation with *Fg*. (D) Wheat spikes at 48 h after inoculation with *Fg*. (E) Wheat spikes at 72 h after inoculation with *Fg*. (F) Wheat spikes at 96 h after inoculation with *Fg*.

**Fig. S2** Nucleotide frequency of the 5ʹ ends of sRNAs in six samples of *Fusarium graminearum* (*Fg*) and infected wheat. (A) Conidia. (B) Mycelia. (C) Wheat spikes at 0 h after inoculation with *Fg*. (D) Wheat spikes at 48 h after inoculation with *Fg*. (E) Wheat spikes at 72 h after inoculation with *Fg*. (F) Wheat spikes at 96 h after inoculation with *Fg*.

**Fig. S3** Clustering analysis of predicted milRNAs in *Fusarium graminearum* (*Fg*). The heatmap shows the 36 *Fg* milRNAs from six samples from conidia, mycelia and wheat spikes 0, 48, 72 and 96 h after inoculation with *Fg*.

**Fig. S4** Schematic diagrams of amino acids from bioH1 and bioH2 and alignment of amino acid sequences of FgbioH1 and FgbioH2 with other bioH members from bacteria. (A) Yellow boxes indicate pimeloyl‐ACP methyl ester carboxylesterase (bioH) domains. (B) The alignment was made using the PROMALS3D multiple sequence and structure alignment server (prodata.swmed.edu/ promals3d/). Representative sequences are coloured according to predicted secondary structures (red: alpha‐helix, blue: beta‐strand). Consensus predicted secondary structure symbols: alpha‐helix, h; beta‐strand, e. Consensus amino acid symbols: conserved amino acids are represented by bold and uppercase letters; aliphatic (I, V, L): l, aromatic (Y, H, W, F): @, hydrophobic (W, F, Y, M, L, I, V, A, C, T, H): h, alcohol (S, T): o, polar residues (D, E, H, K, N, Q, R, S, T): p, tiny (A, G, C, S): t, small (A, G, C, S, V, N, D, T, P): s, bulky residues (E, F, I, K, L, M, Q, R, W, Y): b, positively charged (K, R, H): +, negatively charged (D, E): –, charged (D, E, K, R, H).

**Fig. S5** Southern blot analyses of gene‐deletion strains. For analyses of Δ*FgbioH1* and Δ*FgDicer2* mutant strains, a NEO fragment was used as a probe. For analyses of complementation strains *FgBIOH1C,* a hygromycin fragment was used as a probe.

**Fig. S6** Sanger sequencing chromatograms displaying PPM‐RACE products of large and small fragments including the G‐U junction uncleaved region.

**Fig. S7** Proposed biotin biosynthesis pathway. Numbers in circles indicate three sub‐pathways for the production of two intermediate compounds, pimeloyl‐[ACP] (1 and 2) and pimeloyl‐CoA (3). The dashed arrow represents multiple steps.

**Table S1** Summary statistics of small RNA sequence mapping result (mapping to the *Fusarium graminearum* reference genome).

**Table S2** Summary statistics of sRNA libraries of milRNA.

**Table S3** PCR primers and sequences used in this study.

**Method S1** Experimental procedures.
