# Supplementary material for: Expression of microRNA‐like RNA‐2 (Fgmil‐2) and bioH1 from a single transcript in Fusarium graminearum are inversely correlated to regulate biotin synthesis during vegetative growth and host infection
Source: Mol Plant Pathol. 2019 Aug 6;20(11):1574–81. doi: 10.1111/mpp.12859 (PMC6804420; doi:10.1111/mpp.12859)
Supplement: Supplementary file 2 — Fig. S2 Nucleotide frequency of the 5ʹ ends of sRNAs in six samples of Fusarium graminearum (Fg) and infected wheat. (A) Conidia. (B) Mycelia. (C) Wheat spikes at 0 h after inoculation with Fg. (D) Wheat spikes at 48 h after inoculation with Fg. (E) Wheat spikes at 72 h after inoculation with Fg. (F) Wheat spikes at 96 h after inoculation with Fg. [file MPP-20-1574-s002.docx]

**

**

**Fig. S2** Nucleotide frequency of the 5´ ends of sRNAs in six samples of *F. graminearum* (*Fg*) and wheat
