# Supplementary figures and images for: Expression of microRNA‐like RNA‐2 (Fgmil‐2) and bioH1 from a single transcript in Fusarium graminearum are inversely correlated to regulate biotin synthesis during vegetative growth and host infection
Source: Mol Plant Pathol. 2019 Aug 6;20(11):1574–81. doi: 10.1111/mpp.12859 (PMC6804420; doi:10.1111/mpp.12859)

**
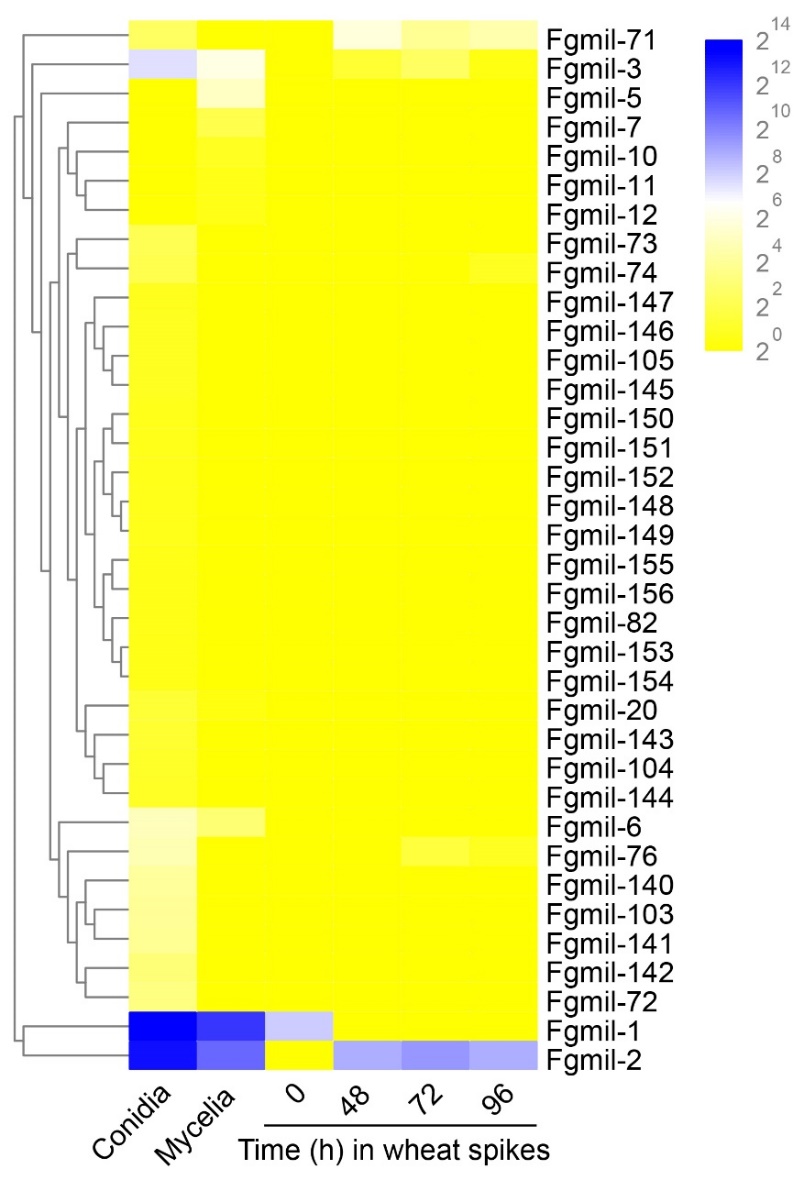
**

**Fig. S3** Clustering analysis of predicted milRNAsin *F. graminearum* (*Fg*)

Supplement: Supplementary file 3 — Fig. S3 Clustering analysis of predicted milRNAs in Fusarium graminearum (Fg). The heatmap shows the 36 Fg milRNAs from six samples from conidia, mycelia and wheat spikes 0, 48, 72 and 96 h after inoculation with Fg. [file MPP-20-1574-s003.docx]

**
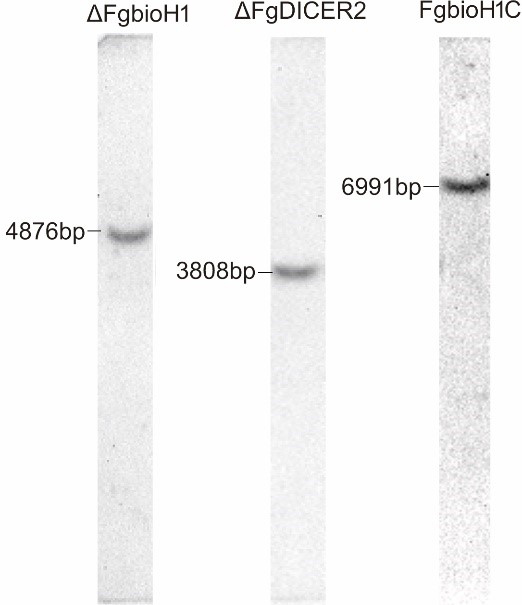
**

**Fig. S5** Southern blot analyses of gene-deletion *F. graminearum* strains

Supplement: Supplementary file 5 — Fig. S5 Southern blot analyses of gene‐deletion strains. For analyses of ΔFgbioH1 and ΔFgDicer2 mutant strains, a NEO fragment was used as a probe. For analyses of complementation strains FgBIOH1C, a hygromycin fragment was used as a probe. [file MPP-20-1574-s005.docx]

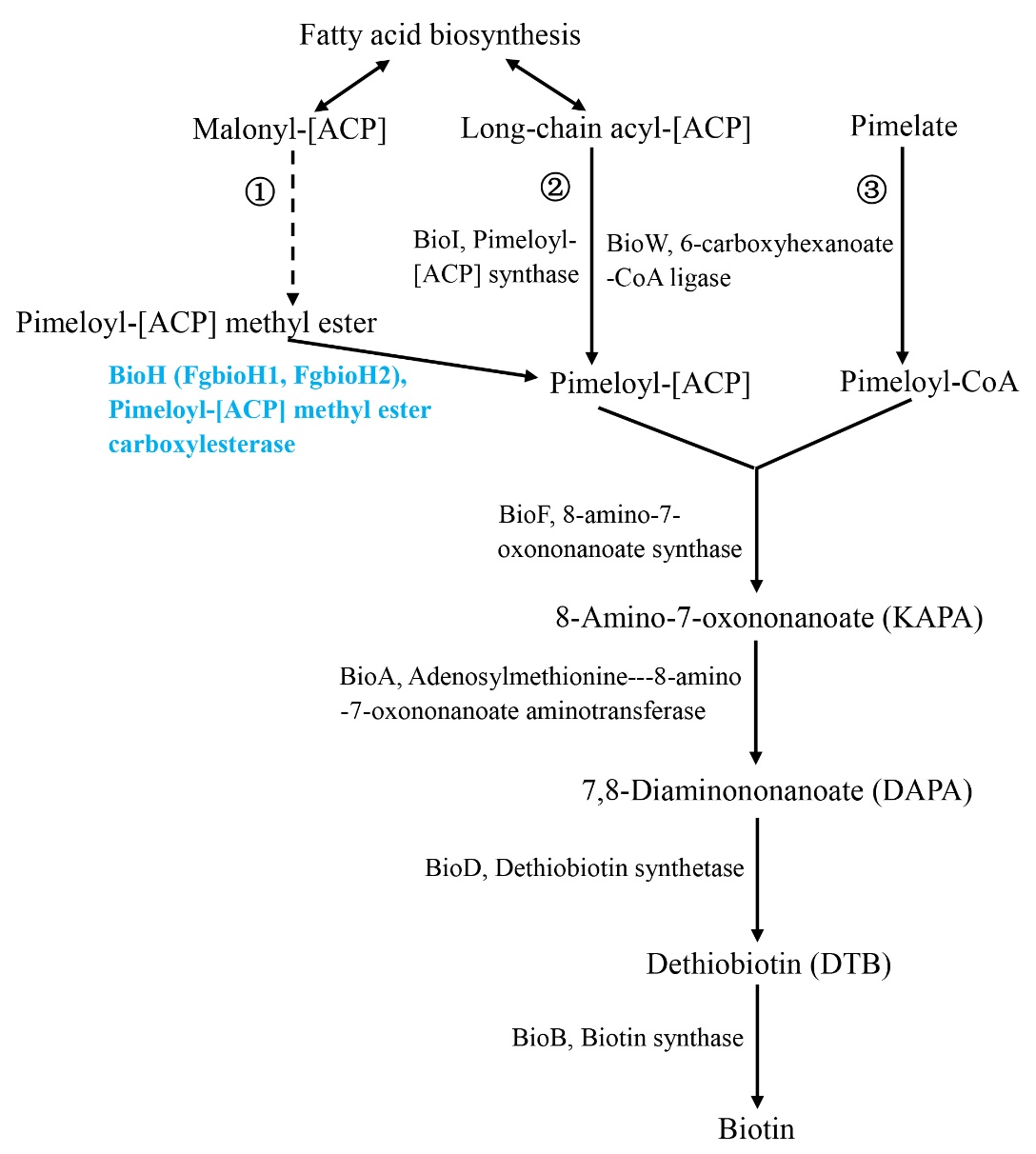


**Fig. S7** Proposed biotin biosynthetic pathway.

Supplement: Supplementary file 7 — Fig. S7 Proposed biotin biosynthesis pathway. Numbers in circles indicate three sub‐pathways for the production of two intermediate compounds, pimeloyl‐[ACP] (1 and 2) and pimeloyl‐CoA (3). The dashed arrow represents multiple steps. [file MPP-20-1574-s007.docx]
