# Supplementary material for: Expression of microRNA‐like RNA‐2 (Fgmil‐2) and bioH1 from a single transcript in Fusarium graminearum are inversely correlated to regulate biotin synthesis during vegetative growth and host infection
Source: Mol Plant Pathol. 2019 Aug 6;20(11):1574–81. doi: 10.1111/mpp.12859 (PMC6804420; doi:10.1111/mpp.12859)
Supplement: Supplementary file 4 — Fig. S4 Schematic diagrams of amino acids from bioH1 and bioH2 and alignment of amino acid sequences of FgbioH1 and FgbioH2 with other bioH members from bacteria. (A) Yellow boxes indicate pimeloyl‐ACP methyl ester carboxylesterase (bioH) domains. (B) The alignment was made using the PROMALS3D multiple sequence and structure alignment server (prodata.swmed.edu/ promals3d/). Representative sequences are coloured according to predicted secondary structures (red: alpha‐helix, blue: beta‐strand). Consensus predicted secondary structure symbols: alpha‐helix, h; beta‐strand, e. Consensus amino acid symbols: conserved amino acids are represented by bold and uppercase letters; aliphatic (I, V, L): l, aromatic (Y, H, W, F): @, hydrophobic (W, F, Y, M, L, I, V, A, C, T, H): h, alcohol (S, T): o, polar residues (D, E, H, K, N, Q, R, S, T): p, tiny (A, G, C, S): t, small (A, G, C, S, V, N, D, T, P): s, bulky residues (E, F, I, K, L, M, Q, R, W, Y): b, positively charged (K, R, H): +, negatively charged (D, E): –, charged (D, E, K, R, H). [file MPP-20-1574-s004.docx]

**
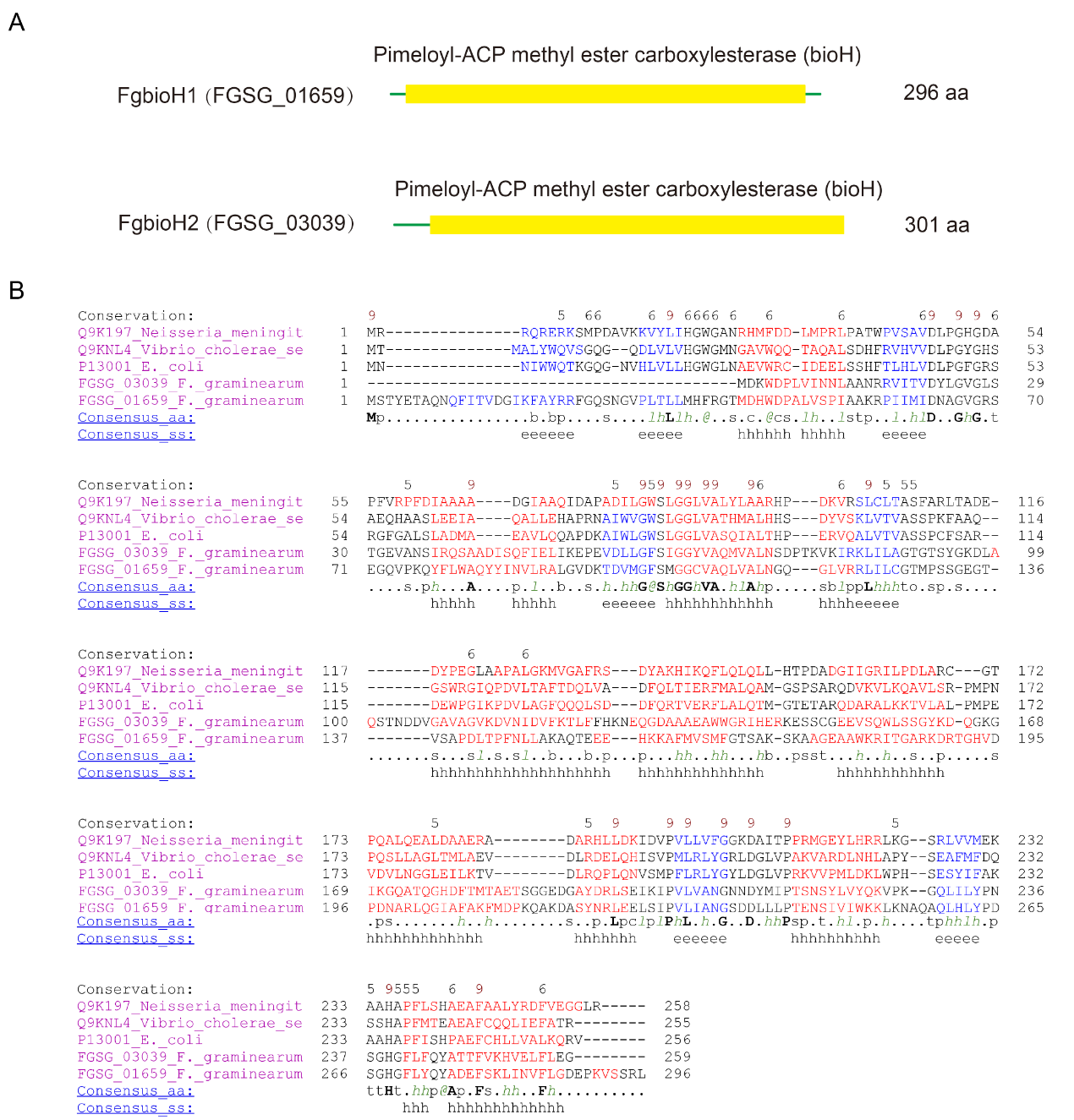
**

**Fig. S4** Schematic diagrams of amino acids from bioH1 and bioH2 and alignment of amino acid sequences of FgbioH1 and FgbioH2 with other bioH members from bacteria
