# Supplementary material for: Expression of microRNA‐like RNA‐2 (Fgmil‐2) and bioH1 from a single transcript in Fusarium graminearum are inversely correlated to regulate biotin synthesis during vegetative growth and host infection
Source: Mol Plant Pathol. 2019 Aug 6;20(11):1574–81. doi: 10.1111/mpp.12859 (PMC6804420; doi:10.1111/mpp.12859)
Supplement: Supplementary file 6 — Fig. S6 Sanger sequencing chromatograms displaying PPM‐RACE products of large and small fragments including the G‐U junction uncleaved region. [file MPP-20-1574-s006.docx]

**
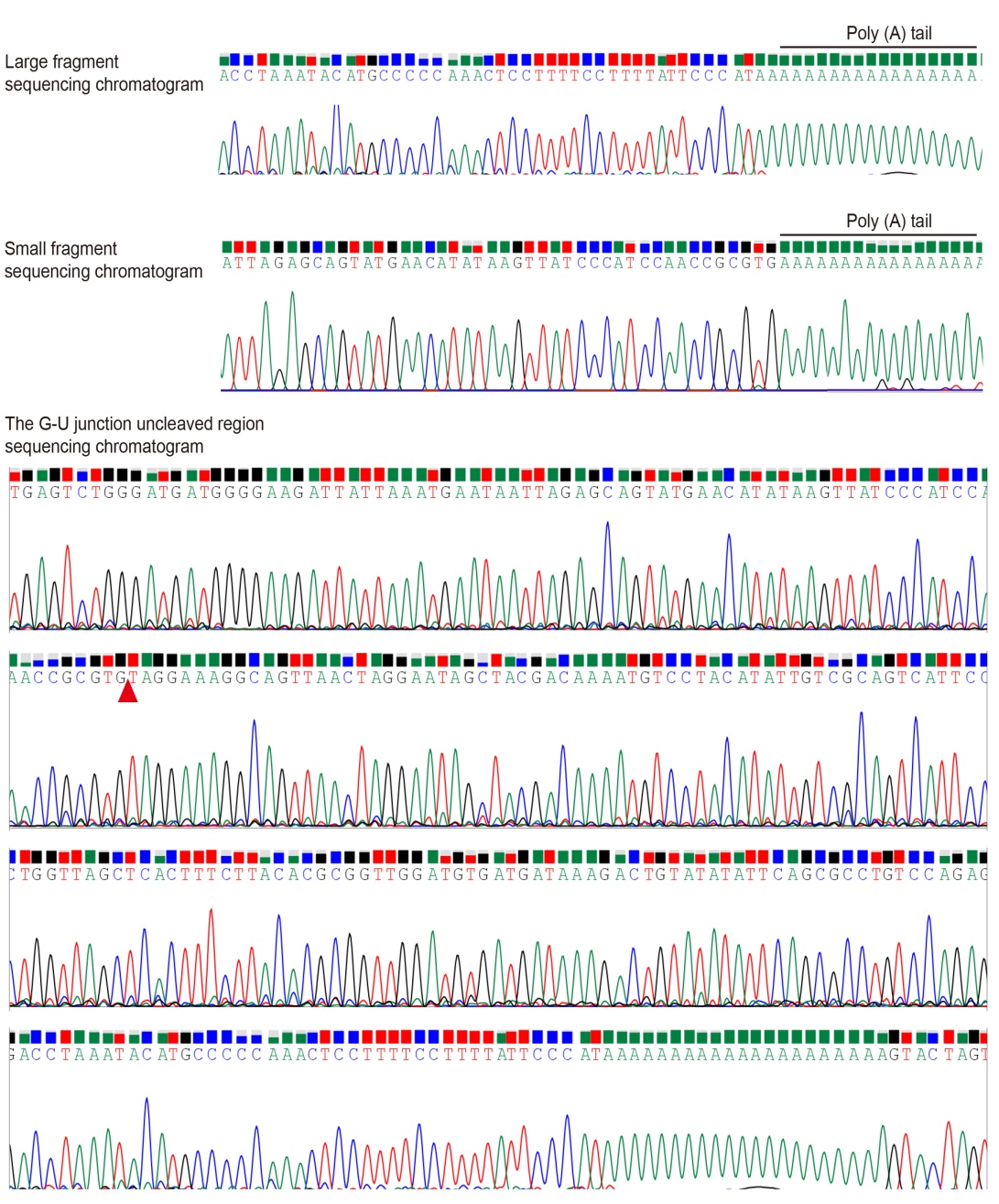
**

**Fig. S6** Sanger sequencing chromatograms displaying PPM-RACE products of large and small fragments including the G-U junction uncleaved region
