# Supplementary material for: Expression of microRNA‐like RNA‐2 (Fgmil‐2) and bioH1 from a single transcript in Fusarium graminearum are inversely correlated to regulate biotin synthesis during vegetative growth and host infection
Source: Mol Plant Pathol. 2019 Aug 6;20(11):1574–81. doi: 10.1111/mpp.12859 (PMC6804420; doi:10.1111/mpp.12859)
Supplement: Supplementary file 8 — Table S1 Summary statistics of small RNA sequence mapping result (mapping to the Fusarium graminearum reference genome). [file MPP-20-1574-s008.docx]

**Table S1** Summary statistics of small RNA sequence mapping results (mapping to the *F. graminearum* reference genome)

| **Library** | **Total reads** | **Perfect match to genome (Total)** | **Percent %**  **(Total)** | **Unique**  **sRNAs** | **Perfect match to genome (Unique)** | **Percent %**  **(Unique)** |
| --- | --- | --- | --- | --- | --- | --- |
| Conidia | 11644892 | 10343665 | 88.83 | 706436 | 370620 | 52.46 |
| Mycelia | 11269468 | 9694283 | 86.02 | 1649716 | 1061490 | 64.34 |
| 0 hai | 23787891 | 97195 | 0.41 | 8083581 | 5687 | 0.07 |
| 48 hai | 23580442 | 635045 | 2.69 | 7248194 | 88272 | 1.22 |
| 72 hai | 22329913 | 2395751 | 10.73 | 3949100 | 160768 | 4.07 |
| 96 hai | 23526957 | 2623485 | 11.15 | 4217318 | 146018 | 3.46 |
