# Supplementary material for: Expression of microRNA‐like RNA‐2 (Fgmil‐2) and bioH1 from a single transcript in Fusarium graminearum are inversely correlated to regulate biotin synthesis during vegetative growth and host infection
Source: Mol Plant Pathol. 2019 Aug 6;20(11):1574–81. doi: 10.1111/mpp.12859 (PMC6804420; doi:10.1111/mpp.12859)
Supplement: Supplementary file 9 — Table S2 Summary statistics of sRNA libraries of milRNA. [file MPP-20-1574-s009.docx]

**Table S2** Summary statistics of sRNA libraries of milRNAs

| ID | MilRNA sequence | Length | Normalized read counts  Transcript per million (TPM) | | | | | |
| --- | --- | --- | --- | --- | --- | --- | --- | --- |
|  |  |  | Conidia | Mycelia | Time (h) in wheat spikes | | | |
|  |  |  |  |  | 0 | 48 | 72 | 96 |
| Fgmil-1 | TCCGGTATGGTGTAGTGGCTA | 21 | 11913.1 | 4359.7 | 277.8 | 0 | 0 | 0 |
| Fgmil-10 | AGTCATAGAAATTAGGGCA | 19 | 0 | 0.825 | 0 | 0 | 0 | 0 |
| Fgmil-103 | TAAGAAAGTGAGCTAACCAGGAA | 23 | 15.0 | 0 | 0 | 0 | 0 | 0 |
| Fgmil-104 | TAAGCAGAACAGAACAAAGAAGAT | 24 | 1.1 | 0 | 0 | 0 | 0 | 0 |
| Fgmil-105 | TGGGTGCTGGACGACGACA | 19 | 0.8 | 0 | 0 | 0 | 0 | 0 |
| Fgmil-11 | TGGATAGCAGGAGGAACCCCAA | 22 | 0 | 0.5 | 0 | 0 | 0 | 0 |
| Fgmil-12 | CGGATTGTAGAGTGCGGCGGA | 21 | 0 | 0.5 | 0 | 0 | 0 | 0 |
| Fgmil-140 | TTCCAGACTGTTAATGCGTTGGCCG | 25 | 16.4 | 0 | 0 | 0 | 0 | 0 |
| Fgmil-141 | GTGGTTATGGGTGGGATACGGGC | 23 | 13.9 | 0 | 0 | 0 | 0 | 0 |
| Fgmil-142 | TCAGAGCGGTGATTGATCAAGA | 22 | 7.8 | 0 | 0 | 0 | 0 | 0 |
| Fgmil-143 | TGGGTACGGGCGGGTTGCGGAA | 22 | 1.5 | 0 | 0 | 0 | 0 | 0 |
| Fgmil-144 | TGACGGTTGGTAGAGGGCGA | 20 | 1.0 | 0 | 0 | 0 | 0 | 0 |
| Fgmil-145 | TGAGAGAGGCTGAGTAGAGA | 20 | 0.8 | 0 | 0 | 0 | 0 | 0 |
| Fgmil-146 | GGTGGAGGAGGAGGAGGAGGAGGA | 24 | 0.8 | 0 | 0 | 0 | 0 | 0 |
| Fgmil-147 | TGGGAGGGTGTGGAGAGGAA | 20 | 0.7 | 0 | 0 | 0 | 0 | 0 |
| Fgmil-148 | TGGGATTGAGGTGGGGGTGGTA | 22 | 0.6 | 0 | 0 | 0 | 0 | 0 |
| Fgmil-149 | TCAGGTTATGAGAGCGCGAA | 20 | 0.6 | 0 | 0 | 0 | 0 | 0 |
| Fgmil-150 | TGCGGTTTATGTGGATCGAA | 20 | 0.6 | 0 | 0 | 0 | 0 | 0 |
| Fgmil-151 | GAGGGAGGCTAGTGGGAAGGAC | 22 | 0.6 | 0 | 0 | 0 | 0 | 0 |
| Fgmil-152 | TTGAGAGTTGATTGGTTTGGTCTA | 24 | 0.6 | 0 | 0 | 0 | 0 | 0 |
| Fgmil-153 | AGGACTGTAGATGGCGTTGGTTTA | 24 | 0.5 | 0 | 0 | 0 | 0 | 0 |
| Fgmil-154 | TAGTAGTACTTTGTAGCTAA | 20 | 0.5 | 0 | 0 | 0 | 0 | 0 |
| Fgmil-155 | GGCAAGAGGAAGGGATGTGGCT | 22 | 0.5 | 0 | 0 | 0 | 0 | 0 |
| Fgmil-156 | TGAGGAGAGTGGCGACGAGAG | 21 | 0.5 | 0 | 0 | 0 | 0 | 0 |
| Fgmil-2 | TAGGAAAGGCAGTTAACTAGGA | 22 | 9032.6 | 1753.3 | 0 | 481.9 | 735.1 | 479.1 |
| Fgmil-20 | TTCGTCCGGGTTGCTTCAA | 19 | 1.7 | 0.3 | 0 | 0 | 0 | 0 |
| Fgmil-3 | GAGGTGGGTTGGGAAGAGGT | 20 | 189.6 | 63.7 | 0 | 1.57 | 5.01 | 0.38 |
| Fgmil-5 | TGGACTAAAGAGCAGAGTACATAT | 24 | 0 | 35.4 | 0 | 0 | 0 | 0 |
| Fgmil-6 | GGTGAGATGGCCGAGTTGGTTA | 22 | 30.6 | 7.3 | 0 | 0 | 0 | 0 |
| Fgmil-7 | AAGGGTGGTGGTTGAGAATGAT | 22 | 0 | 3.0 | 0 | 0 | 0 | 0 |
| Fgmil-71 | GGTACTGTGGTCTAGTTGGT | 20 | 4.9 | 0 | 0 | 55.1 | 13.8 | 23.3 |
| Fgmil-72 | GGCGCAGTGGCAGAGTGGTCTA | 22 | 9.7 | 0 | 0 | 0 | 0 | 0 |
| Fgmil-73 | CGGATAGGAGAAAGACACACGAGCA | 25 | 3.6 | 0 | 0 | 0 | 0 | 0 |
| Fgmil-74 | CTGGACTAAAGAGCAGAGTACATAT | 25 | 3.0 | 0 | 0 | 0 | 0 | 0.8 |
| Fgmil-76 | GTCCTTCGGACTCAGCAGGCT | 21 | 26.4 | 0 | 0 | 0 | 2.1 | 0.8 |
| Fgmil-82 | AAAGCGATTCAATTCATCTTTT | 22 | 0.5 | 0 | 0 | 0 | 0 | 0 |
