# Supplementary material for: Expression of microRNA‐like RNA‐2 (Fgmil‐2) and bioH1 from a single transcript in Fusarium graminearum are inversely correlated to regulate biotin synthesis during vegetative growth and host infection
Source: Mol Plant Pathol. 2019 Aug 6;20(11):1574–81. doi: 10.1111/mpp.12859 (PMC6804420; doi:10.1111/mpp.12859)
Supplement: Supplementary file 10 — Table S3 PCR primers and sequences used in this study. [file MPP-20-1574-s010.docx]

**Table S3** PCR primers and sequences used in this study

| Primer | Sequence (5’-3’) | Relevant characteristics |
| --- | --- | --- |
| bioP1 | GTTGATTTTGTAGGTTTAGAGT | PCR primers to amplify *FgbioH1* upstream fragment for the construction of *FgbioH1* deletion mutants |
| bioP2 | agccacgattcgaagccgcgATAAAAAACAAAATCACAGTCATGTCCC |  |
| bioP3 | catgcatgttgcatgatgatCTATGCAAATGTTTTGGCATCTTAGAGCTGT | PCR primers to amplify *FgbioH1* downstream fragment for the construction of *FgbioH1* deletion mutants |
| bioP4 | GATAGGCGCATGAGGTGGATTTC |  |
| bio-UF | GGTGAACAACAACCCAAGTCC | PCR primers for identification of *FgbioH1* deletion transformants |
| bio-DR | GCAAGAACACGGACAAGAAGG |  |
| dicP1 | TGTCTCGACCTTTGATATCCTGCGA | PCR primers to amplify *FgDicer2* upstream fragment for the construction of *FgDicer2* deletion mutants |
| dicP2 | agccacgattcgaagccgcgTCGTAGATGTTTTGCTTTCGAATCTTGGTG |  |
| dicP3 | catgcatgttgcatgatgatCGGTACCCTTCGGCGAAAGCATT | PCR primers to amplify *FgDicer2* downstream fragment for the construction of *FgDicer2* deletion mutants |
| dicP4 | CTCTTCCAAAGGCTCCATCGTGGG |  |
| dic-UF | CTGCTACAGGCGAGAAGGAGA | PCR primers for identification of *FgDicer2* deletion transformants |
| dic-DR | GTCGGTCTTTGAAATGGTTGAG |  |
| bioc-P1 | aaggaaaaaagcggccgcCTTTCCAGGGGCTGATTGTC | PCR primers to amplify the native promoter region and open reading frame of *FgbioH1* |
| bioc-P2 | ccttaattaaTCATAGACGGCTAGACACCTTGGGTTCATCACC |  |
| bioc-UR | AGAGGGAAGAGCCAAAGACG | PCR primers for identification of *BIOH1C* upstream insertion site |
| tetP1 | CGTTAGGGTTTGGTGGTTCC |  |
|  |  |  |
| hgro-DF | CCAATACGAGGTCGCCAACA | PCR primers for identification of *BIOH1C* downstream insertion site |
| tetP4 | TCAAAGCAACGGTCAAAGGA |  |
| bio-GSP | ACGAGACCGCCCAAAACCAG | PCR primers to amplify *FgbioH1* 3´UTR for the 3´RACE PCR |
| bio-nestGSP | CATCACTGTAGATGGCATCA |  |
| tubulin-P1 | ATGGTCCTCGATCTCCTTTGTTGA | RT-PCR primers to quantify the mRNA expression level of housekeeping gene *tubulin* |
| tubulin-P2 | TGGTCGGATTTGCCCCTCTG |  |
| bio-qP1 | CGCCGCTAAGCGACCTAT | RT-PCR primers to quantify the mRNA expression level of *FgbioH1* |
| bio-qP2 | TCCTCCCATTGAGAAACCCA |  |
| dic-qP1 | GAGGAACTCGCCAAACTAGCA | RT-PCR primers to quantify the mRNA expression level of *FgDicer2* |
| dic- qP2 | CTTCTCTGCACGTTTCTGCTCC |  |
| neoP1 | CGCGGCTTCGAATCGTGGCT | PCR primers for amplification of neomycin resistance gene(*NEO*) |
| neoP2 | ATCATCATGCAACATGCATG |  |
| BPL-qP1  BPL-qP2 | GCAATTTACGCTCAGTTTCTCC  TTCAACATTCCCCAATCACG | RT-PCR primers to quantify the mRNA expression level of *BPL* |
| DUR1,2-qP1 | AAGACTTGCTTTTGCCGTATCA | RT-PCR primers to quantify the mRNA expression level of *Dur1,2p* |
| DUR1,2-qP2 | CATCGTCGCCGTCACTCG |  |
| ACC-qP1 | TTCATCTACATCCCTCCCTTCG | RT-PCR primers to quantify the mRNA expression level of *ACC* |
| ACC-qP2 | CAATCATACCCTCGGGCTCA |  |
| PC-qP1 | CTGGAAACTCGATTCCTGGTAC | RT-PCR primers to quantify the mRNA expression level of *PC* |
| PC-qP2 | CTGCTGTTGTCCTGCTGCTT |  |
| PCCA-qP1 | ACCAAGGTCACTGTCTTTCAGC | RT-PCR primers to quantify the mRNA expression level of *PCCA* |
| PCCA-qP2 | GCACCCTTTTGCACCGTTT |  |
| Fgmil-1  Fgmil-2  Fgmil-3 | TAGCCACTACACCATACCGGA  TCCTAGTTAACTGCCTTTCCTA  ACCTCTTCCCAACCCACCTC | The sequences of antisense RNA Northern probes |
