# Supplementary material for: Expression of microRNA‐like RNA‐2 (Fgmil‐2) and bioH1 from a single transcript in Fusarium graminearum are inversely correlated to regulate biotin synthesis during vegetative growth and host infection
Source: Mol Plant Pathol. 2019 Aug 6;20(11):1574–81. doi: 10.1111/mpp.12859 (PMC6804420; doi:10.1111/mpp.12859)
Supplement: Supplementary file 11 — Method S1 Experimental procedures. [file MPP-20-1574-s011.docx]

**Method S1 Experimental procedures**

**Strains and growth conditions**

*Fg* strain 5035, isolated from a scabby wheat spike in Wuhan, China (Qu *et al.*, 2008), was used for experiments and production of derivative strains. Fungal strains were cultured at 28°C on potato dextrose agar (PDA) and broth (PDB) for mycelium growth, and in CMC broth for conidiation (Duvick *et al.*, 1992). Gene-deletion strains, Δ*FgDicer2* and Δ*FgbioH1*, were selected on PDA plates plus G418 (30 μg ml^-1^), while hygromycin B (100 μg ml^-1^) and G418 (30 μg ml^-1^) were used for selection of *FgBIOH1C*. Minimal medium (MM) (Duvick *et al.*, 1992) was used for culture of *Fg* strains at 28°C for 5 days in the absence and presence of biotin (20 ng ml^-l^, Sigma-Aldrich, MO, USA) for gene expression assays; to measure biotin content, all strains were cultured in MM broth and incubated at 28°C for 3 days with shaking (200 rpm).

**Small RNA libraries for sequencing**

Total RNA was extracted using Trizol reagent (Invitrogen, CA, USA). Small RNAs (sRNAs) (18 to 30 bp) were isolated from a 15% denaturing poly-acrylamide gel. 5´adaptor and 3´adaptor were incubated with each small RNA sample in the presence of T4-RNA ligase, and the adapted RNAs were reversely transcribed into cDNAs (Weiberg *et al.*, 2013) to construct sRNA libraries. The libraries were sequenced using the Illumina HiSeq 2500 platform (BGI, Wuhan, China).

**Small RNA sequencing analysis**

Raw sequencing reads were filtered by removal of adaptors, poor quality reads, and contaminants. Using SOAP (<http://soap.genomics.org.cn>), clean reads that matched the *Fg* genome, identified in the FungiDB website (http://fungidb.org/common/downloads/release-3.0/Fgraminearum_PH-1/fasta/data/) were used for study, while reads matching the *Triticum aestivum* genome from the Ensembl website (<ftp://ftp.ensemblgenomes.org/pub/plants/release-26/fasta/> triticum_aestivum) were excluded. To eliminate known, non-coding structural RNAs, such as rRNA, tRNA, snRNA, and snoRNA, all sequences were aligned to Rfam 10.1 (http://rfam.janelia.org/) and Genbank database using BLAST (<ftp://ftp.ncbi.nlm.nih.gov/genbank/>). Novel milRNAs and their secondary structures were identified from unannotated sRNAs using MIREAP ([https://sourceforge.net/ projects/mireap](https://sourceforge.net/%20projects/mireap)).

**Generation of gene-deletion mutant and complementation strains**

Gene-deletion mutant and complementation strains were generated as previously described (Song *et al.*, 2016). An upstream fragment (988 bp) and a downstream fragment (969 bp) of *FgbioH1* were amplified from *Fg* 5035 by PCR with the bioP1/bioP2, and bioP3/bioP4 primer pairs, while a Neomycin resistance gene (Neo) (1427 bp) was amplified from the pNeo vector (Song *et al.*, 2016) by PCR with one primer pair of neoP1/neoP2. All primers used in this study are listed in Table S3 (see Supporting Information). These three fragments, flanking the Neo gene, were fused by overlap extension PCR (SOE-PCR) with the bioP1/bioP4 primer pair, to generate a 3384 bp fragment that was used to construct the *FgbioH1* gene-deletion vector pMD18-KObioH1. Similarly, an upstream fragment (988 bp) and a downstream fragment (912 bp) flanking the *FgDicer2* gene were amplified by PCR with dicP1/dicP2 and dicP3/dicP4 primer pairs. The fragments were then used for SOE-PCR with the Neo gene amplified by dicP1/dicP4 primers to generate a 3327 bp fragment for construction of the *FgDicer2* gene-deletion vector pMD18-KOdicer2.

To generate a complementation strain *FgBIOH1C*, a 2707 bp fragment containing the native *FgbioH1* promoter and coding sequence was amplified by PCR and inserted into the NotI - PacI site of the pHgro vector to make the pHgro-BIOH1C vector. This vector was then introduced to a *PLS1* site of the Δ*FgbioH1* mutant strain using *Fg* protoplasts for transformation (Song *et al.*, 2016).

**Southern blot and Northern blot hybridization**

Southern blot was performed as previously described (Song *et al*., 2016). For Northern blot hybridization, total RNA was isolated from conidia and mycelia and wheat spikes at 0, 48, 72 and 96 h after inoculation with *Fg* 5035. Thirty μg of total RNA for each sample were electrophoresed on a 15% denaturing polyacrylamide gel and transferred onto a Hybond-NX membrane (GE Healthcare, NJ, USA) as previously described (Kim *et al*., 2010). A microRNA Marker (New England Biolabs, MA, USA) was used as a size marker. The ethidium bromide stained rRNA bands in the polyacrylamide gel show equal loading of RNA. Oligonucleotide for complementary sequences of *Fg* milRNAs were used as Northern hybridization probes and labelled by digoxigenin-11-dUTP (Roche, IN, USA) following the supplier’s instructions and the probe sequences are in Table S3.

**Quantitative RT-PCR**

Total RNA isolated as described above was treated with ribonuclease-free DNaseI (Fermentas, St Leon-Rot, Germany) at 37°C for 30 mins. Aliquots (4 μg) of the treated RNA were converted to cDNA using a RevertAid First Strand cDNA Synthesis Kit with an oligo(dT)_18_ primer (Fermentas, St Leon-Rot). Real-time quantitative PCR (qPCR) was run on a Lightcycler Roche 480 with LightCycler 480 SYBR Green I Master kit (Roche, IN, USA). An *Fg* tubulin gene was co-amplified as an internal reference. Each qPCR assay was carried out in three replicates and the 2^–ΔΔCT^ method of relative gene quantification (Livak & Schmittgen, 2001) was used to calculate the expression level of respective genes relative to the reference.

**Poly(A) polymerase-mediated rapid amplification of cDNA at 3´ends**

To map *FgDicer2*-mediated cleavage products and identify the 3´-UTR of *FgbioH1* transcripts, polymerase-mediated rapid amplification of cDNA at 3´ends were performed as previously described (Wang, 2015). Poly(A) tailing of total RNA from *Fg* 5035 and the *FgDicer2* strain was conducted using *E. coli* poly (A) polymerase (New England Biolabs, MA, USA). 3´RACE was carried out using Rapid Amplification of cDNA Ends kit (Invitrogen, CA, USA).

**Determination of biotin content**

Biotin extraction and HPLC (High-performance liquid chromatography) analysis were carried out as previously described (Höller *et al*., 2006) for determination of biotin contents from *Fg* strains. One milliliter of conidia (5×10^5^ spores ml^-1^) was added to one liter of MM broth and cultured as above. Mycelia collected through miracloth were washed with sterile distilled water and lyophilized. One gram of lyophilized mycelia was ground in liquid nitrogen and extracted for analysis of biotin content.

**Virulence assay**

Wheat (*cv*. X76) spikes were inoculated and incubated in a plastic house as previously described (Song *et al.*, 2016). Infected spikelets were scored and photographed at 14 dai. Percentages of infected spikelets were calculated as means ± SD of 25 spikes for each strain.

**Mycotoxin determination**

Mycotoxin and ergosterol contents from wheat spikes at 21 dai were extracted and determined using gas chromatography–mass spectrometry (GC-MS) as previously described (Song *et al.*, 2016).

**References**

**Duvick, J.P., Rood, T., Rao, A.G. and Marshak, D.R.** (1992) Purification and characterization of a novel antimicrobial peptide from maize (*Zea mays L*.) kernels. *J Biol Chem*. **267,** 18814-18820.

**Höller, U., Wachter, F., Wehrli, C. and Fizet, C.** (2006) Quantification of biotin in feed, food, tablets, and premixes using HPLC–MS/MS. *J Chromatogr B Analyt Technol Biomed Life Sci*. **831,** 8-16.

**Kim, S.W., Li, Z., Moore, P.S., Monaghan, A.P., Chang, Y., Nichols, M. and John, B.** (2010) A sensitive non-radioactive northern blot method to detect small RNAs. *Nucleic Acids Res*. **38,** e98.

**Livak, K.J. and Schmittgen, T.D.** (2001) Analysis of relative gene expression data using real-time quantitative PCR and the 2^−ΔΔCT^ method. *Methods*. **25,** 402-408.

**Qu, B., Li, H.P., Zhang, J.B., Xu, Y.B., Huang, T., Wu, A.B., Zhao, C.S., Carter, J., Nicholson, P. and Liao, Y.C.** (2008) Geographic distribution and genetic diversity of *Fusarium graminearum* and *F. asiaticum* on wheat spikes throughout China. *Plant Pathology*. **57,** 15-24.

**Song, X.S., Xing, S., Li, H.P., Zhang, J.B., Qu, B., Jiang, J.H., Fan, C., Yang. P., Liu, J.L., Hu, Z.Q., Xue, S. and Liao, Y.C.** (2016) An antibody that confers plant disease resistance targets a membrane-bound glyoxal oxidase in *Fusarium*. *New Phytol*. **210,** 997-1010.

**Wang, C. and Fang, J.** (2015) RLM-RACE, PPM-RACE, and qRT-PCR: an integrated strategy to accurately validate miRNA target genes. *Methods Mol Biol*. **1296,** 175-186**.**

**Weiberg, A., Wang, M., Lin, F.-M., Zhao, H., Zhang, Z., Kaloshian, I., Huang, H.D. and Jin, H.** (2013) Fungal small RNAs suppress plant immunity by hijacking host RNA interference pathways. *Science*. **342,** 118-123.
